# A novel therapeutic anticancer property of raw garlic extract via injection but not ingestion

**DOI:** 10.1038/s41420-018-0122-x

**Published:** 2018-11-21

**Authors:** Zhiming Li, Wenjun Le, Zheng Cui

**Affiliations:** 1 Department of Pathology, Wake Forest University Health Sciences, Winston-Salem, 27157 NC USA; 2 Family Planning Research Institute, Center of Reproductive Medicine, Tongji College, Huazhong University of Science and Technology, Wuhan430030, Hubei, China; 3 The Institute for Translational Nanomedicine, Shanghai East Hospital, Tongji University School of Medicine, Shanghai, 200120 China

## Abstract

Prior studies suggest a possibility that the anticancer property of garlic is more effective only when exposed directly to cancer cells than absorbed first by the normal epithelial cells of the gastrointestinal tract wall. We tested this possibility in two mouse models of highly aggressive malignancies that cannot yet be cured by conventional therapies: sarcoma 180- and EL4-induced lethal ascites. Daily oral gavages of raw garlic extract (RGE; equivalent to 100 mg wet weight) for 21 days failed to offer any meaningful effect in the mice with malignancies. However, the daily injection of the same amounts of the same materials for 21 days completely cured all the mice of cancer. This novel anticancer activity of RGE was present entirely in the size fraction of the molecules smaller than 3000 Dalton rather than the larger molecules and was completely partitioned into the organic phase rather than into the aqueous phase. One half of the anticancer activity was inactivated by heating at 100 °C for 10 min, suggesting that multiple components were concertedly involved. In a direct comparison, the RGE was significantly more effective in killing the cultured cancer cells in vitro than the extracts from other 21 raw vegetables and fruits. In cell culture, RGE killed a wide variety of different cancer cells regardless of species of origin and cell types. Cancer cells generally are well known to be defective in many common metabolic pathways present in their normal cell counterpart for processing normal nutrients. The metabolism of these otherwise normal nutrients could be stalled in the cancer cells and become cytotoxic. The most-effective way of treating cancer by RGE may be the direct injection instead of eating the cooked garlic.

## Introduction

For over several thousand years, garlic has been consumed by humans not only as a kind of flavoring food but also as a medicinal food or a topical agent. As a medicinal food, raw or cooked garlic has been used to treat infections^1–7^, to lower cholesterols^8–11^ and to inhibit the formation of blood clots, etc.^12–14^. The antibacterial properties of garlic were first described by Pasteur as early as in 1858. The topical applications of the raw garlic pastes were widely used as anti-infection agents in the WWI and WWII by the soldiers^15,16^. In the recent decades, the anticancer properties of garlic have also been extensively studied in cell cultures, in animals, and in humans.

Most anticancer studies in humans were the retrospective survey to determine whether there were possible connections between the consumption of the cooked garlic and cancer incidents or slower progression^17–21^. Some studies were the intervention studies by feeding the human subjects with garlic. But direct evidence supporting an anticancer contention of garlic was weak^22,23^. The compounds extracted from the garlic, especially the sulfuric compounds, have also been shown to mildly reduce the incident rates and severity of the tumor formation induced by the administrated *N*-nitroso compounds in animal models^24^. The reductionist view also led to many studies of the individual compounds isolated from garlic for their anticancer properties. In most animal studies, when garlic or the purified garlic compounds were given via ingestion, the direct anticancer effects were weak at best^25^.

There are also many studies suggesting that garlic may have direct anticancer properties in cell culture. For an example, in a large study of 34 different vegetable juices against eight different human cancer cell lines, raw garlic extract (RGE) when added directly to the cultured cancer cells stood out as the most-effective anticancer agent in comparison with all other 33 raw vegetable extracts^26^. Furthermore, this cytotoxic effect was highly specific against cancer cells but not the non-cancerous cells^27,28^. This highly selective anticancer cytotoxicity without harming normal cells was also in agreement with the commonly known fact that garlic is a food and can be consumed safely in large quantities without significant adverse side-effect. Similar anticancer properties of garlic in cell culture were also reported by other studies using different cancer cells^29,30^. Despite the efforts in the last several decades, the anticancer effects of garlic still lack a piece of convincing evidence: a decisively curative result against aggressive cancer in the animal models or in humans.

There is also an apparent difference in anticancer effects when garlic is cooked or not and exposed to cancer cells directly or going through gastrointestinal (GI) tract first. In this study using a couple of mouse cancer models that are rapidly lethal and have not been cured by any conventional therapy, we tested the hypothesis that the direct exposure of RGE to cancer cells via injection may significantly improve its anticancer effect than ingestion in which it has to be absorbed and processed by normal cells.

## Results

### Curative effect of RGE ip injection against EL4-induced ascites

**Fig. 1:**
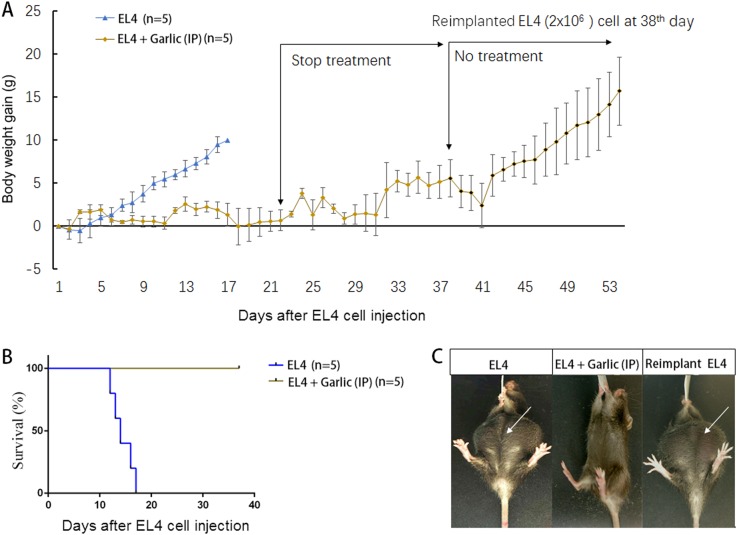
The therapeutic activity of RGE injection against EL4-induced ascites. Ten C57BL6 mice were inoculated ip with 2 × 10^6^ EL4 (mouse ascites lymphoma cells). One day later, five of these mice were treated daily by the ip injection of 1 ml RGE (equivalent to 100 mg wet weight of raw garlic) between day 1 and day 22. The treatment was stopped for all the five mice between day 23 and day 37. The mice were re-injected with 2 × 10^6^ EL4 ip and left untreated until moribund. **a** The body weight gain in grams for reflection of ascites development. **b** Survival curves of the mice in different groups. **c** Representative images of the mice with and without ascites

EL4 is a line of highly aggressive mouse lymphoma cells that induces rapid and lethal ascites in mice as described previously^31^. In this experiment, all the syngeneic C57BL/6 mice were given intraperitoneal (ip) injection with 2 × 10^6^ EL4 cells from the cell culture. Twenty-four hours later, the injected mice were divided into the groups of RGE ip injection as treatment and the control without RGE injection. For the treated group, each mouse of average 15 g body weight was given an ip injection of 1 ml RGE that was equivalent to 100 mg wet weight of raw garlic. The control group was left untreated. The ip injection of RGE was repeated daily for 21 days. As shown in Fig. 1, all eight mice in the control group, as expected, developed ascites that increased the body weight significantly higher than the mice without ascites (Fig. 1a). The ascites development was markedly visible (Fig. 1b, c). All the mice in the control group were moribund as indicated in the figure and were killed. However, the mice given the ip injection of RGE were healthy without any sign of adverse effect (Fig. 1b, c). Moreover, when the injection of RGE stopped between days 22 and 37 (or longer up to four more weeks, data not shown), the mice remained healthy, indicating there was no re-growth of ascites even long after the RGE treatment stopped. To assure that the failure to develop ascites in these mice was solely due to the garlic treatment and not due to other unknown factors, on day 38 all the mice of the previously RGE treated group were again given the ip injection of 2 × 10^6^ EL4 cells and left without the injection of RGE. Re-challenge with EL4 cells developed ascites in all the mice (Fig. 1a), indicating that the failure of ascites development was entirely due to the RGE treatment.

### Curative effect of RGE ip injection, not ingestion, against S180-induced ascites

**Fig. 2:**
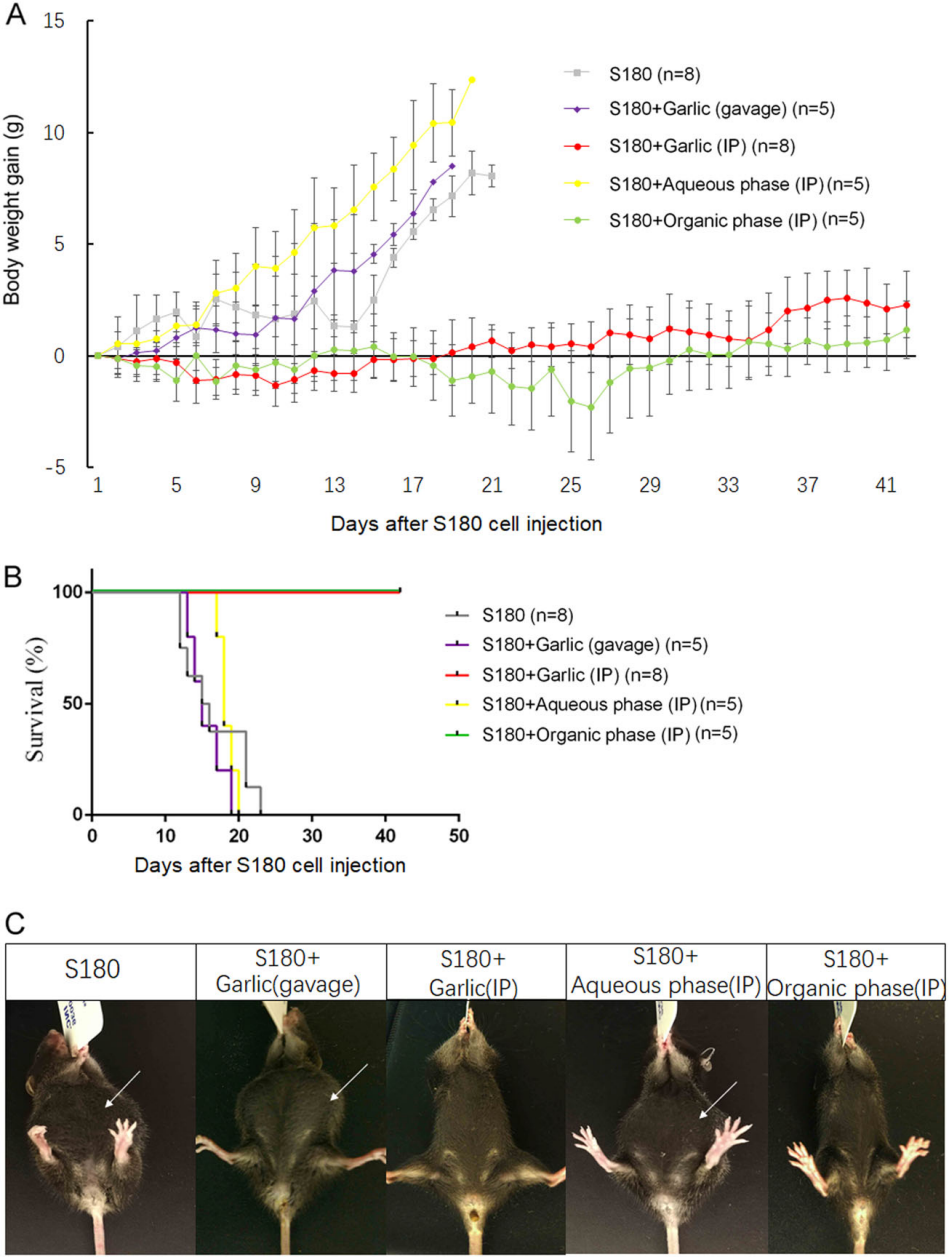
The therapeutic activity of RGE injection against S180-induced ascites. Thirty-one C57BL6 mice were inoculated ip with 2 × 10^6^ S180 cells (mouse sarcoma cells). One day later, five mice were given 1 ml RGE via daily oral gavage between day 1 and day 22. Eight mice were treated by the ip injection of 1 ml RGE (equivalent to 100 mg wet weight of raw garlic) daily between day 1 and day 22. Five mice were treated by the ip injection of 1 ml aqueous phase of RGE (equivalent to 100 mg wet weight of raw garlic) daily between day 1 and day 22. Five mice were treated by the ip injection of 1 ml organic phase of RGE (equivalent to 100 mg wet weight of raw garlic) daily between day 1 and day 22. **a** The body weight gain in grams for reflection of ascites development. **b** Survival curves of the mice in different groups. **c** Representative images of the mice with and without ascites

S180 is another line of mouse aggressive lethal sarcoma cell. The survival time (average 15 days) was slightly longer than that of EL4 (average 13 days) when the same doses were given ip. All the mice were given ip injection with a dose of 2 × 10^6^ S180 cells. One day later, the mice were divided into five different groups. Three groups were treated daily with three different preparations of RGE: RGE, the aqueous phase and the organic phase of RGE, at a dose equivalent to 100 mg of the original raw garlic wet weight. One group was given RGE orally via gavages with the daily doses equivalent to 100 mg wet weight of garlic for each mouse. One group was left untreated as the control. As expected, all the mice in the control group developed ascites and were euthanized after moribund (Fig. 2a, b, c). Similarly, all the mice in the group treated with RGE given via gavages developed ascites without any increase in the survival time. Moreover, all the mice in the group treated with the aqueous phase of RGE given via ip also developed ascites without any significant increase in the survival time. In a sharp contrast, when RGE or the organic phase of RGE was given ip at a same daily dose for 21 days, all the mice in these two groups remained healthy without any sign of adverse effect.

### Comparison of RGE with the extracts from other fruits and vegetables for their anticancer activities

**Fig. 3:**
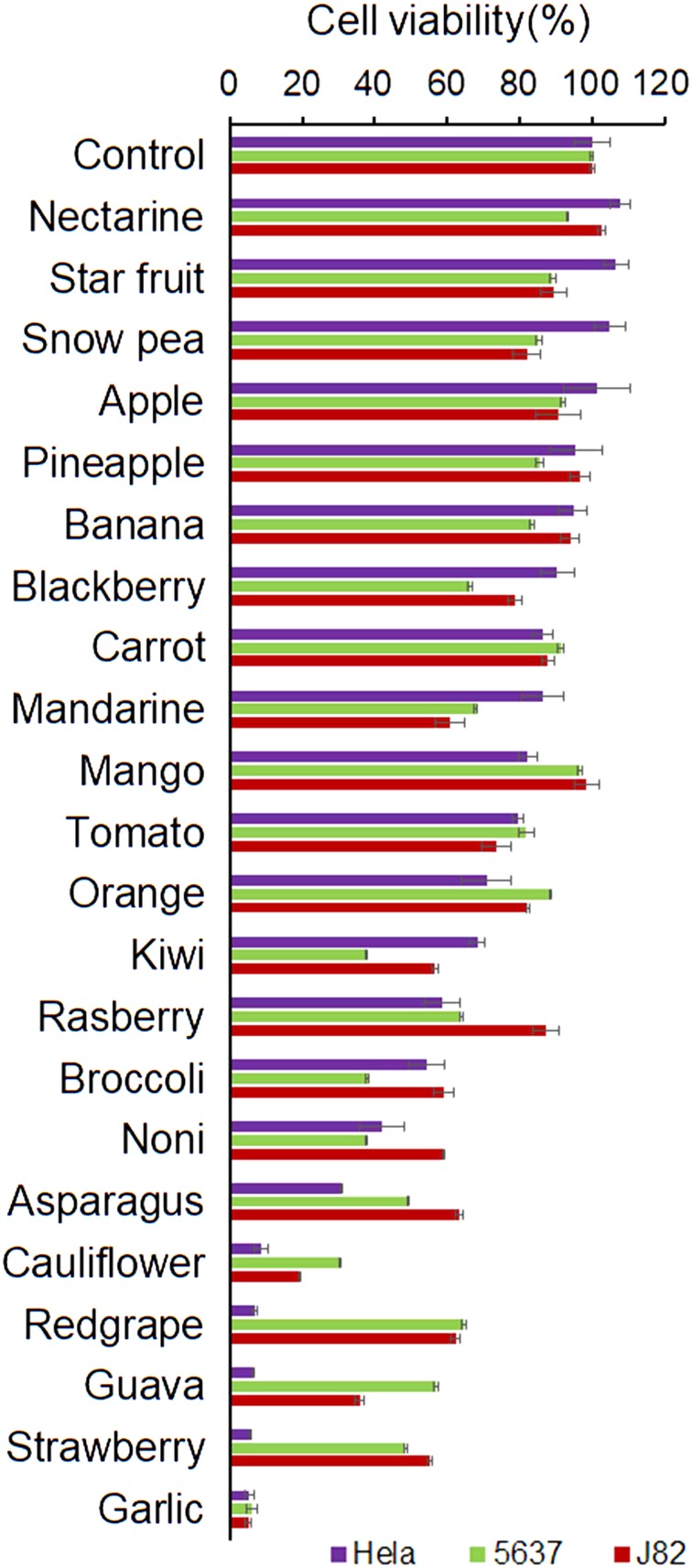
In vitro killing of human cancer cells by co-culturing with the extracts of different fruits and vegetables. All extracts of fruits and vegetables were prepared in the same manner and similarly diluted to 1/200 of their respective wet weights (5 mg/ml) for co-culturing with Hela (human cervical carcinoma) and human bladder cancer cells 5637 and J82. After 24 h in the cell culture condition at 37 °C, the percentages of survived cancer cells were measured by CCK-8

To compare the anticancer property of RGE, we prepared extracts of additional seven vegetables and 14 fruits in the way similar to that of RGE preparation. All the dilution factors were normalized to their respective original wet weights. All the extracts were added individually to the cell cultures of three different human cancer cell lines for direct comparison: Hela (human cervical carcinoma), 5637 (human bladder carcinoma), and J82 (human bladder adenocarcinoma). The cancer cells were plated at 15% confluence and grew for 24 h at 37 °C before the juices were added to a 1/200 dilution factor of their respective wet weights (5 mg/1 ml). The pH values of the final solutions were unaffected as the volumes of the added extracts were relatively small and the culture medium color indicator of pH remained unchanged till the end of cell culture. The cells were co-cultured for another 24 h at 37 °C before the viable cells in each well were analyzed with CCK-8 kit. As seen in Fig. 3, RGE was the only one that killed all three lines of cancer cells completely. We also tested RGE against additional cancer cells and found that it was equally effective in killing all other cancer cells tested so far, including but not limited to EL4 and S180 (data not shown). The anticancer property of RGE was in agreement to the previous study on eight cancer cell lines (PC-3, AGS, U-87, DAOY, MCF-7, A-549, Panc-1, and Caki-2) at a much higher concentration (166 mg/ml)^26^. RGE was also shown to be highly effective in killing above eight cancer cell lines 100%. In addition, the extracts from cauliflower, red grape, guava, and strawberry exhibited equal effectiveness against Hela cells but much less-killing activity against 5637 and J82 (Fig. 3). RGE had stronger anticancer property against various types of cancer cells in cell cultures in comparison with the extracts from other vegetables and fruits tested so far.

### Size fractionation of the RGE anticancer activity

**Fig. 4:**
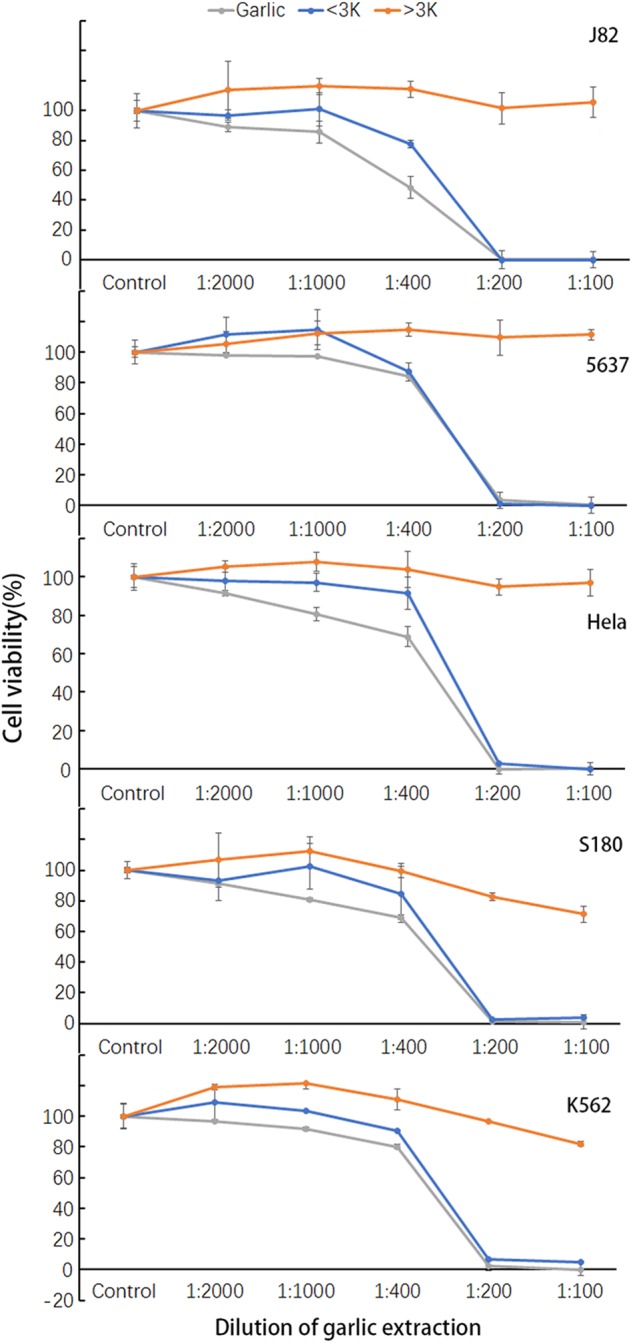
Distribution of cancer cell killing activity in the size fractions of RGE. RGE was fractionated with a size filter membrane of 3000 Dalton cutoff into the fraction smaller than 3000 Dalton (< 3 K) and the fraction larger than 3000 Dalton (> 3 K). The fractions were co-cultured with five different kinds of cancer cells at 37 °C for 24 h at the indicated dilutions. Survived cells were measured by CCK-8

To roughly estimate the size(s) of the active molecules involving the anticancer property, we partitioned RGE through a molecular size filter, via which the molecules smaller than 3000 Dalton went through the membrane and the molecules larger than 3000 Dalton were retained at the top chamber of the filter. After centrifugation, most yellowish color and viscosity remained in the top fraction, whereas the lower fraction was clear and not viscous. The fractions were brought back to the original volumes by distilled water. The size fractions and RGE were added to the cell cultures of five different cancer cell lines and co-cultured for 24 h at 37 °C. Viable cells were analyzed by CCK-8 kit. The results in Fig. 4 show that the anticancer property of the ≤ 3K fraction was nearly identical to that of RGE at all the dilutions against all five cancer cell lines. On the other hand, the anticancer property was completely absent from the ≥ 3 fraction against the same cell lines. In addition, the 1/200 dilution (5 mg/ml) seemed to be a critical concentration, via which the least amount of garlic achieved the maximum effect.

### Solubility fractionation of the anticancer activity in RGE

**Fig. 5:**
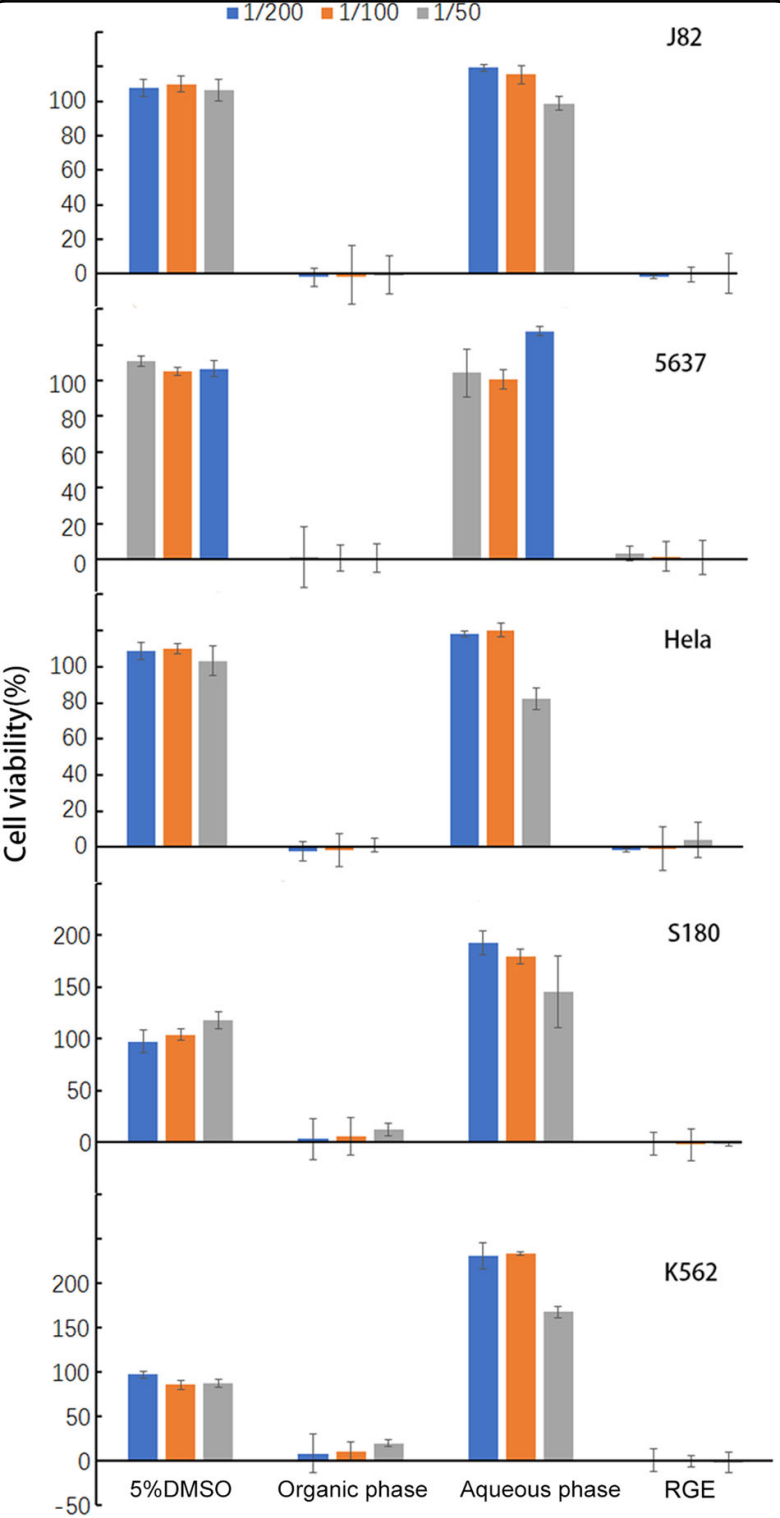
In vitro killing of five different cancer cell lines by aqueous phase and organic phase of RGE. The aqueous phase and organic phase of RGE were prepared as described in the section of methods. After co-culturing with the indicated fractions of RGE at 37 °C for 24 h, survived cells were measured by CCK-8

To determine the solubility of the anticancer property, we partitioned RGE into the organic phase and aqueous phase via chloroform extraction. The aqueous phase was brought back to the original volume by phosphate-buffered saline (PBS) and the organic phase was reconstituted back to the original volume by 5% dimethyl sulfoxide (DMSO). At the indicated dilutions, the different RGE preparations were co-cultured individually with five different cancer cell lines for 24 h. As shown in Fig. 5, all anticancer properties of RGE were present in the organic phase. Instead of having the anticancer property, the aqueous phase of RGE even stimulated the growth of S180 and especially K562 human leukemia cells to onefold more than RGE.

### RGE induced apoptosis in cancer cells

**Fig. 6:**
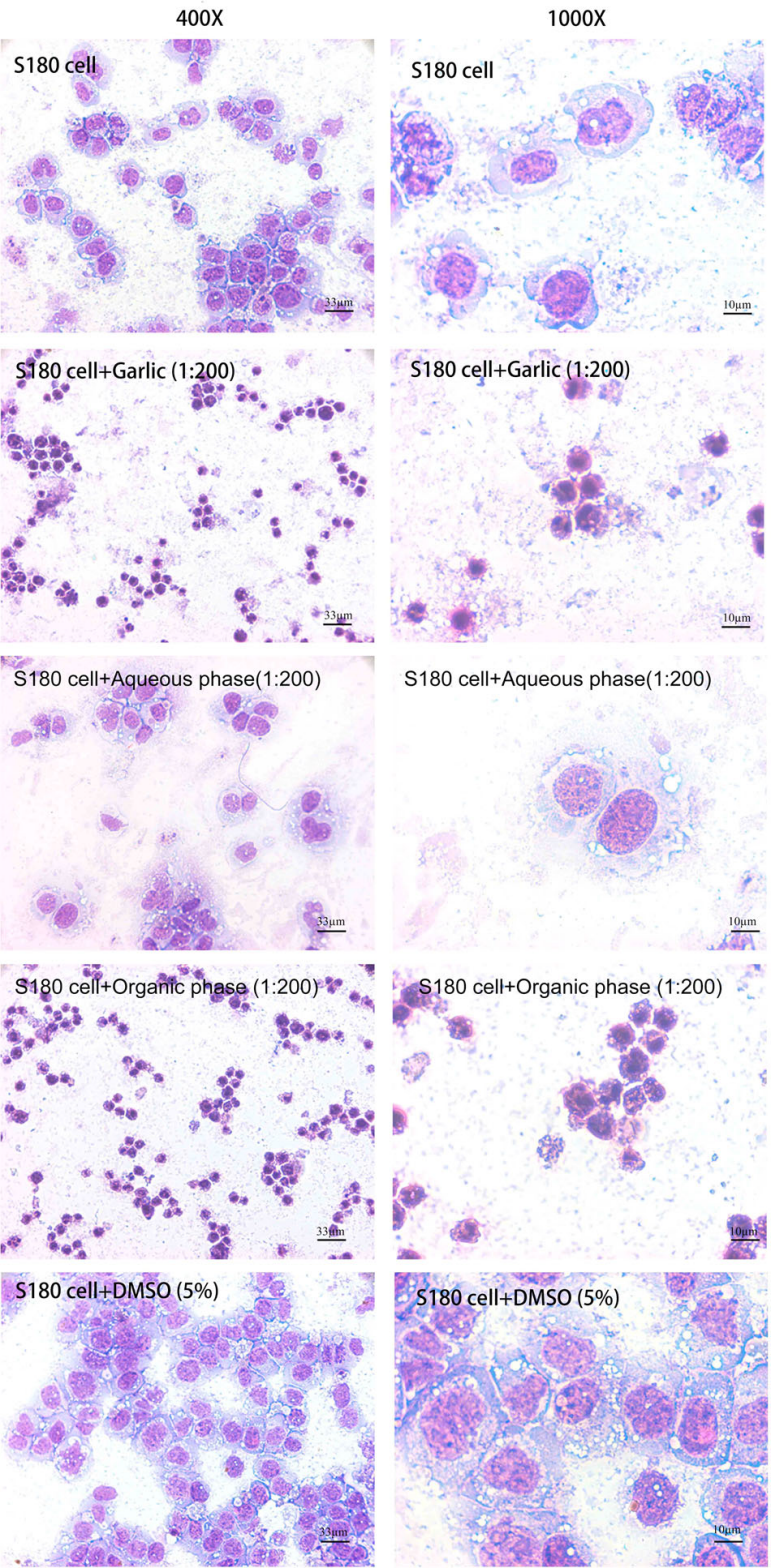
Morphologies of the S180 cells treated by RGE and its different fractions of hydrophobicity. The aqueous phase and organic phase of RGE were prepared as described in the methods section. After co-culturing with the indicated fractions of RGE at 37 °C for 24 h, cells were spun down onto glass slides, stained with Diff-Quik and examined and photographed with light microscope

To examine the mechanisms of the induced cancer cell death, S180 cells after being treated by various RGE preparations were examined morphologically. After treated by RGE or the organic phase, all S180 cells showed the highly condensed cellular and nucleus morphology, being consistent with apoptosis (Fig. 6). However, S180 cells treated with the aqueous phase or 5% DMSO did not show any sign of cell death.

### Partial inactivation of RGE anticancer activity by heat

**Fig. 7:**
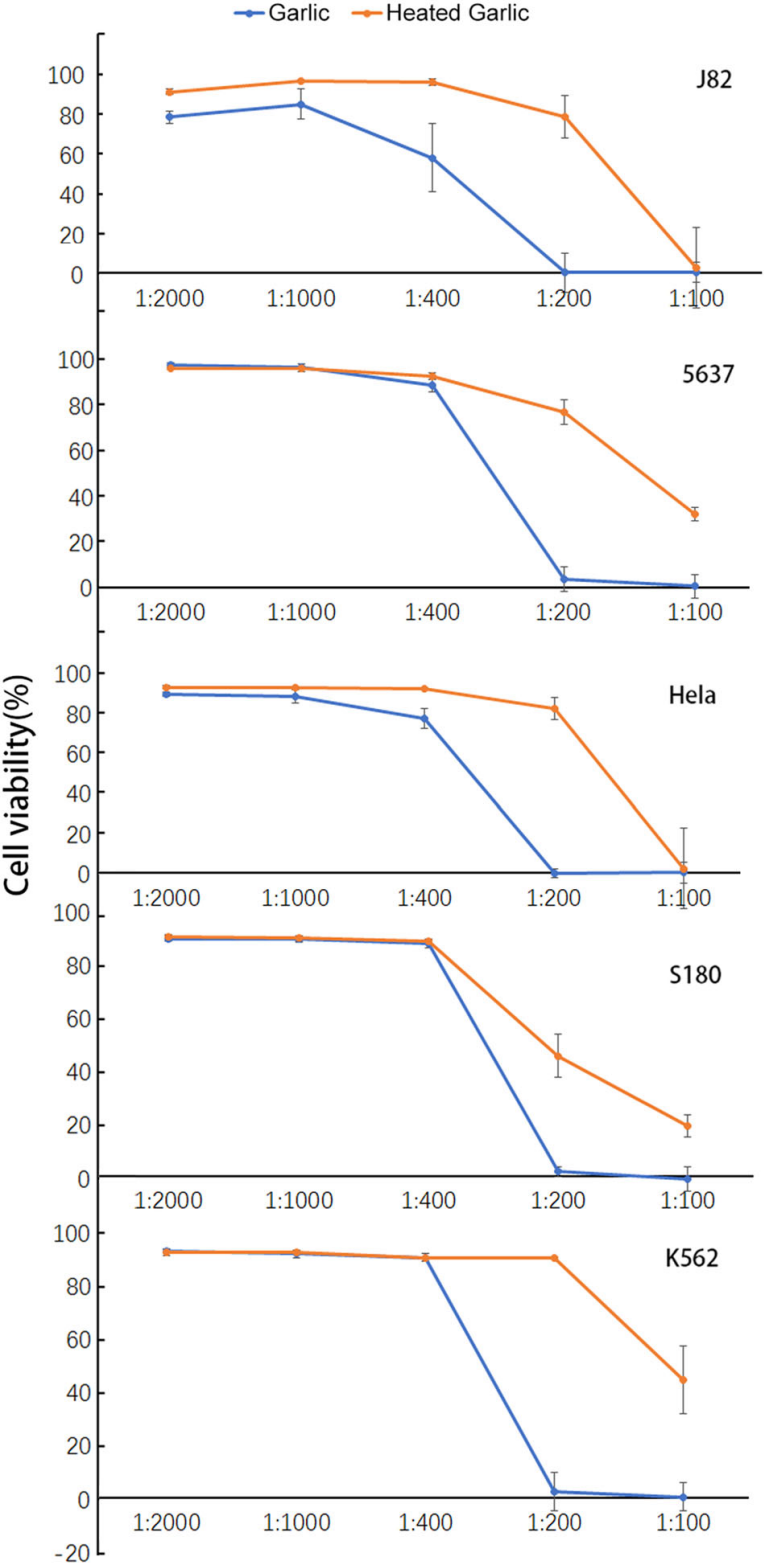
Heat sensitivity of RGE anticancer activity against five different cancer cell lines. The heated RGE was heated in a metal heating block at 100 °C for 10 min. After co-culturing with RGE or the heated RGE at 37 °C for 24 h, the survived cells were measured by CCK-8

As a flavoring food, garlic is often ingested after cooking. Many studies of the anticancer properties were also done with the cooked garlic. To determine its sensitivity to heat, we heated RGE at 100 °C for 10 min. As shown in Fig. 7, the heat-treated RGE was compared with the unheated RGE for their anticancer properties against five different cancer cell lines. At 1/100 dilution, the heat treatment did not alter the anticancer activity of RGE against J82 and Hela, but reduced the anticancer properties partially against 5637, S180, and K562. At 1/200 dilution, the cancer killing activities against J82, 5637, Hela, and K562 were mostly, and against S180 was partially, abolished by the heat treatment. These results indicate that some anticancer activities of RGE were heat-labile and others were heat-resistant.

### High selective killing of RGE against cancer cells but not normal cells

**Fig. 8:**
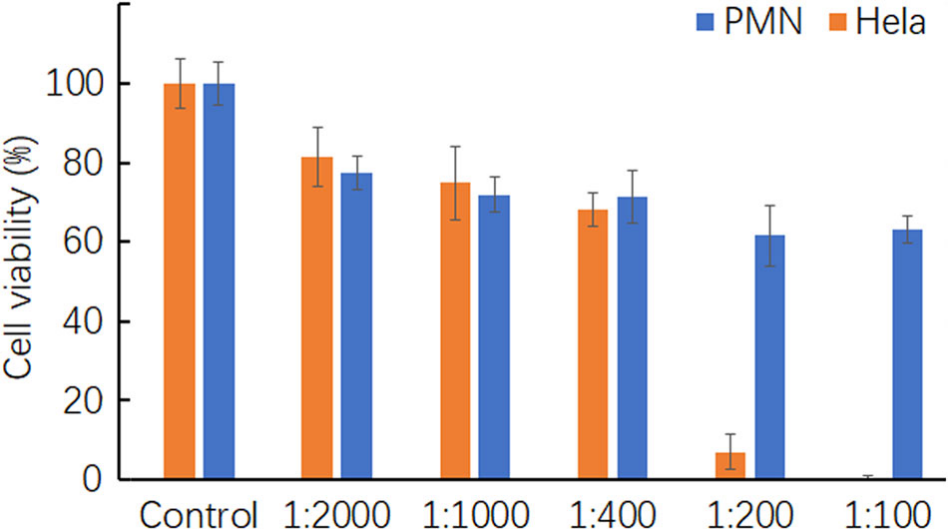
Selectivity of RGE in killing cancer cells but not normal cells. Cell viability in cancer and normal cells was examined by CCK-8 assay after 24 h of incubation at 37 °C with RGE

Our results showed that the injected RGE had no adverse effect on the treated animals, indicating that RGE did not harm normal cells. We wanted to further verify this ability of RGE to distinguish cancer cells from normal cells in the in vitro system. We isolated human polymorphic nucleus (PMN) cells from the blood of healthy donors. Human PMN and human cervical carcinoma Hela cells were co-cultured with RGE at different dilutions. At 1:400 or higher dilutions of RGE, there was no specific cytotoxicity against PMN or Hela (Fig. 8). At 1:200 or lower dilutions nearly all Hela cells were killed and most PMN were alive. The apparent selectivity against cells in vitro was in an agreement to that of the in vivo results of the injected RGE.

## Discussion

The major finding of our study is that RGE had a novel therapeutic effect against even the most aggressive models of malignancy in mice only when injected. Ingestion of the same amount of RGE failed to offer any meaningful effect on the same model of malignancy. This novel anticancer activity in the mouse models was also recapitulated in the cell culture in which the direct cancer cell killing by RGE stood out far better than the extract from any other 21 vegetables and fruits. This activity was contributable to the multiple garlic compounds that were partially heat-sensitive, organic-soluble and smaller than 3000 Dalton. The treated mice did not display any adverse side-effect in terms of behavior and physical appearance from receiving daily injections of RGE. Necropsy also failed to find any abnormality in any organ and tissue of the treated mice either from the malignancies or from the injected RGE.

One novel aspect of the injected RGE was its ability to cure the most aggressive mouse cancer models that cannot be cured by any conventional therapy. Historically, there have been two major types of modeling cancer in mice for drug testing. The most popular model is to transplant human cancer cells into the mice, to form visible nodules, and to test the effects of drugs on the nodule sizes. The other model, although rarely used nowadays, was to transplant aggressive cancer cells that induce rapid lethality and to test the effects of agents on the survival of the mice. So far, there has not been any report of any conventional therapy to be successful in curing this type of the induced mouse malignancy and effectively rescued the mice from death. The two mouse models used in this study represented this second type of malignancy with the extreme aggressiveness in which the survival times, if treated unsuccessfully, are between 13 and 15 days. The mice in these types of malignancy underwent cachexia, internal bleeding, and the failure of major organs that, unlike the first model, represent all the terminal events of human cancer deaths. Therefore, the ability of the injected RGE to completely kill all the cancer cells without any adverse side-effect in mice may represent one of the most potent anticancer properties reported so far, giving the hopes of significant beneficial effects if being used in humans.

Another surprising aspect of our findings was the complete opposite effects of RGE injection in comparison with ingestion. This novel finding showed that the extremely potent anticancer activity of RGE was only achieved by direct exposure to cancer cells and was completely lost by going first through the GI tract. Given the fact that garlic is a flavoring food, one can safely reason that all the compounds in garlic are the unharmful nutrients to the normal cells and can be taken even in large quantity by humans without adverse side-effect. Ingestion is a process in which all the nutrients in the food must be absorbed first into the epithelial cells of the GI tract walls. Before being released into the bloodstream, some nutrients have to be processed or modified metabolically in the epithelial cells. This process apparently destroyed all the anticancer property of the ingested RGE in addition to the possibility that the digestive enzymes and acidity in the cavities of stomach and intestines may also be destructive to the anticancer activity. Although very simple, the profound anticancer property of the injected RGE has not been reported in the literature. Nearly, all the garlic studies were done with the ingested garlic or injections of individually isolated garlic compounds. However, injection of raw food juice or parenteral nutrients to cancer patients who are still capable of normal eating is rarely practiced in the clinical settings. The anticancer therapeutic potential of normal nutrients from fruits and vegetables without being first processed by the intestinal epithelial cells may have never been tested. Yet, this study also showed that many other raw fruits and vegetables also had significant anticancer properties when directly exposed to cancer cells. Therefore, injection of raw food juices may represent a new concept of treatment and may provide novel therapeutic potentials in comparison to ingestion of food after cooking.

Our results also showed that the killing of cancer cells but not normal cells by RGE was highly specific. This selective killing of cancer cells was also reflected in the treated mice that had neither visible physical side-effect nor abnormality in any organ or tissue upon necropsy. This selectivity must be based on the intrinsic yet common differences between many, if not all, cancer cells and normal cells. It is possible that cancer cells may gain some unique metabolic properties for being targeted uniquely by the garlic compounds. But so far the experimental data is rare in supporting such a scenario. Some gain-of-function changes of cancer cells have been identified. But however, none of these changed functions was exclusive to cancer cells as normal cells also have them only at somewhat lowered levels. For example, all cancer cells show highly elevated cell division that also shared by some normal cells. The non-selective cytotoxic effects on the rapidly growing hair follicle cells and GI tract epithelial cells by many chemotherapy compounds are the reasons for hair loss and bad GI tract reactions. To establish such a gain-of-function targeting mechanism for the selectivity, one has to first find such a novel target that is present only in all cancer cells and is absent from all normal cells. So far, no such a cancer marker matches all these criteria.

However, an opposite scenario for such an absolute selectivity is highly feasible. We would like to propose one possible mechanism to explain the selective cancer killing. The garlic compounds may target one or more loss-of-function pathways in cancer cells. In these cases, the nutrients that can be easily processed metabolically in the normal cells cannot be processed in the cancer cells owing to their defective pathways of metabolism. Any nutrient that can be up-taken by the cells but cannot be processed metabolically would be accumulated intracellularly and become cytotoxic. Cancer cells are well known to be the metabolically streamlined cells in which many metabolic pathways become defective in comparison to their normal cell counterparts. For example, nearly all cancer cells are defective in pathways related to mitochondria functions^32,33^. In addition, hepatoma cells of rat or human are also defective in lipid methylation pathways^34–36^.

Other vegetables and fruits, such as cauliflower, red grape, guava, and strawberry also showed similar anticancer properties, but, unlike RGE, the extents of their activities varied from cancer cell lines to lines. Nevertheless, we believe that the extracts from these raw vegetables and fruits may also work better via injection than ingestion for their anticancer activities. There is no indication so far that these activities were additive when the different extracts were combined together. It also came to our attention that RGE produced from different geological regions may have different potencies of anticancer property. It is not clear whether the differences were due to differences in subspecies of garlic or the soil qualities for the garlic growth. Further investigations seem to be needed.

The identification of the active compounds in RGE is underway. But it may be complicated by the fact that the activity may involve multiple compounds concertedly in RGE. However, with or without identification of specific active compounds first, RGE as a food can be used safely in animals and humans as the parenteral nutrients via intravenous or ip injection without the concern of cytotoxicity to normal cells. Although microbes cannot survive in RGE, it is reasonable to filter the preparations through the 0.22 µm filters to remove any potential live microbes before injection.

## Materials and methods

### Preparation of garlic and other fruit and vegetable extracts

All 22 fruits and vegetables, including raw garlic, were purchased from the local supermarket of Winston-Salem, North Carolina, USA. The cloves of fresh raw garlic or other fruits and vegetables were weighted and mixed with distilled water at a ratio of 1:9 (gram:ml) before being blended at the top speed for 3 min with a 1100-watt Ninja Professional 1100 Kitchen Blender. The blended juices were poured through a cheese cloth to filter out large debris and centrifuged at 500 g at 4 °C for 30 min to remove small debris yielding clear extracts. These extracts were stored in aliquots at − 80 °C till further uses. All the dilution factors in the final solutions were calculated by the folds of dilution from the original wet weights of the individual fruits and vegetables.

### Size separation of RGE

A portion of the garlic extracts was centrifuged through a spin-filter unit (Amicon Ultra-0.5 ml, 3 k; Millipore) at 14,000 × *g* for 30 min. After centrifugation, the filtrates that passed through the membrane (the < 3 k fraction) and the fluid that stayed above the membrane (the ≥ 3 k fraction) were collected, respectively.

### Solubility separation of organic RGE

For the separation based on hydrophobicity, an equal volume of chloroform/methanol (1:1) was added to RGE. After thorough homogenization, an equal volume of 1-butanol/50 mM NaCl (4:5, v/v) mixture was added before vortexing and sonicating till being completely mixed. The tubes were centrifuged in a swing-bucket rotor with 350 × *g* for 10 min at the room temperature. After centrifugation, RGE was separated into three phases: a colorless upper aqueous phase; a white solid interphase; and a light-yellowish lower organic phase. The aqueous phase and organic phase were collected carefully. The aqueous phase was dried with a gentle stream of nitrogen gas to remove the methanol and brought up with PBS to the original volume before phase separation. The organic phase was completely dried under a flow of nitrogen and brought up with 5% DMSO to the original volume before phase separation.

### Cell lines

J82 and 5637 human bladder cancer cells, Hela human cervix cancer cell, K562 human myelogenous leukemia cell, EL4 murine musculus lymphoma cell, and S180 murine sarcoma cell were purchase from American Type Tissue Culture Collection. J82 and Hela cells were maintained in minimum essential medium with 10% fetal bovine serum (FBS). EL4, S180, and K562 cells were maintained in DMEM with 10% FBS. 5637 was maintained in RPMI 1640 with 10% FBS. The cultures were incubated at 37 °C with humidified 5% CO_2_. C57BL/6 mice (Charles River Breeding Laboratories) were housed under the controlled conditions (temperature 25 °C, light cycle 12 h).

### Malignant ascites in mice

Malignant ascites was induced in C57BL/6 mice by an ip injection of 2 × 10^6^ S180 or EL4 cell suspension in 1 ml PBS as previously described. Twenty-four hours after the cancer cell ip injection (day 0), mice were divided into the groups of the designed treatments and untreated controls. The mice were inspected and weighted daily for the assessment of ascites development. All of the animal research procedures were approved by the Institutional Animal Care and Use Committee.

### Cytotoxicity assay

Cancer cells were seeded in the 96-well plates to ~ 20% confluency and grew for 24 h at 37 °C and all experimental groups were done in triplicates. After incubations with various juices and preparations for 24 h at 37 °C, the viable cells in each well were determined using the Cell Counting Kit-8 (CCK-8, Enzo Life Sciences) according to the manufacturers’ instruction. In brief, one-tenth volume of CCK-8 was added to each well and incubated for 30 min at 37 °C. The 450-nm absorbance was measured in an automatic microplate reader. Untreated cells were used as the controls for 100% cell viability.

### Microscopic analysis of cell morphology

After various treatments with RGE and other preparations, S180 cells were collected from the wells and cytospun onto glass slides and stained with Protocol Hema-3 (Fisher Diagnostics), an eosin/hematoxylin staining. Cellular morphology was analyzed and photographed under the light microscope.

### Statistical analysis

Statistical analysis of the results was performed with a two-sided, unpaired Student’s *t* test with a significance level of 5%. The survival percentages were performed with GraphPad Prism version 5 (GraphPad Software, San Diego, CA)
